# Persistent and emerging district-level hotspots of domestic violence against women in India: a spatiotemporal analysis, 2020–2024

**DOI:** 10.3389/fpubh.2026.1994555

**Published:** 2026-10-29

**Authors:** K. Susmitha, J. Balamurugan, Upali Weerakoon

**Affiliations:** 1 Department of Social Sciences, School of Social Sciences & Languages, Vellore Institute of Technology, Vellore, India; 2 Department of Sociology, Faculty of Arts, University of Peradeniya, Peradeniya, Sri Lanka

**Keywords:** crime against women, domestic violence, hotspot analysis, India, public health surveillance, spatial epidemiology

## Abstract

**Introduction:**

Domestic violence (DV) against women remains a substantial public health concern in India. However, most existing spatial analyses of crime against women examine a single crime, a single state or region, or a short study period.

**Methods:**

This study conducted a district-level spatiotemporal analysis of recorded domestic violence against women across Indian districts between 2020 and 2024. Dowry Deaths, Abetment to Suicide, Cruelty by Husband or Relatives, Dowry Prohibition Act offenses, and Protection of Women from Domestic Violence Act cases were selected from the National Crime Records Bureau’s crime categories and integrated into a composite standardized Domestic Violence Index (DV Index). District-level population estimates were derived from Census 2011 boundaries and official population projections. Global Moran’s I and Local Getis-Ord Gi* statistics were used to assess spatial autocorrelation and identify significant hotspots for each study year. Mann–Kendall trend statistics were used to classify persistent, recurrent, and directional (new, emerging, intensifying, and diminishing) hotspot typologies.

**Results:**

Global Moran’s I indicated statistically significant spatial autocorrelation in the DV Index in every study year (0.36–0.53, all *p* ≤ 0.0001), with the highest value in 2024. This indicated that spatial clustering increased overall, although not continuously, across the study period. Local analysis identified 30 districts (4.7%) as persistent hotspots and 52 (8.2%) as recurrent hotspots, concentrated mainly in a belt across northern and central India, with a smaller cluster in Telangana. Directional analysis identified 31 emerging and 38 intensifying hotspot districts. Hotspot identification was largely stable under a *k*-nearest-neighbors spatial weights specification and moderately stable under an alternative weighting scheme; however, the strict persistent hotspot classification did not survive false discovery rate correction.

**Discussion:**

Because the index is based on police-recorded cases, these hotspots show where formal registration of domestic violence is concentrated, not necessarily where prevalence or unmet need is highest. Read alongside information on reporting capacity and survey-based prevalence, such maps can help prioritize protective and health-system services.

## 1 Introduction

Violence against women, including intimate partner violence and domestic violence (DV), is not only a private or criminal justice issue (1). It is a major public health problem with consequences for women’s physical, mental, sexual, and reproductive health, requiring coordinated responses from health, justice, and social service sectors (2). The Declaration on the Elimination of Violence Against Women (3) formally defines such violence as gender-based acts that cause, or are likely to cause, physical, sexual, or psychological harm occurring within the family, the wider community, or through state action or inaction. The World Health Organization’s (WHO) ecological framework for violence prevention identifies interacting individual, relationship, social, cultural, and environmental factors as drivers of violence (1). In India, domestic violence against women has been documented as a substantial and persistent public health and human rights concern for decades. Household survey evidence has found domestic violence to be pervasive across caste, class, religion, age, and education groups (4). Fewer than 2% of women experiencing domestic violence had approached the police to file a complaint (5), providing early evidence of the wide gap between women’s lived experience of violence and its formal registration. Informal support systems were limited because neighbors and family members commonly treated violence as a private matter (4). This gap remains pronounced in nationally representative surveys such as the National Family Health Surveys (NFHS). According to NFHS-5, 32% of ever-married women had experienced physical, sexual, or emotional violence from their spouse, yet only 14% of women sought help to stop it (6). By contrast, *Crime in India*, published by the National Crime Records Bureau (NCRB), recorded 66.4 cases per 100,000 women in 2022, 66.2 in 2023, and 64.6 in 2024 (7–9). This rate covers all registered crimes against women, yet it remains far below survey-based prevalence estimates (10). This underscores that police-recorded data capture formal system engagement with domestic violence rather than its full underlying burden. Cruelty by Husband or Relatives has consistently constituted the largest single category of registered crime against women nationally throughout this period (7–9).

India’s legal framework addresses domestic violence through several distinct instruments, including provisions relating to Dowry Deaths and Cruelty by Husband or Relatives (11), the Dowry Prohibition Act (1961) (12), and the Protection of Women from Domestic Violence Act (PWDVA) (2005) (13). Multivariate spatial analyses of crime against women in Uttar Pradesh and Maharashtra found only modest spatial correlations between categories such as rape and Dowry Deaths (14) and distinct spatial gradients for domestic cruelty relative to other forms of violence against women (15). This suggests that each category may be shaped by partially distinct local conditions, including demographic factors such as skewed sex ratio, which has itself been directly linked to an elevated risk of criminal victimization among women (16–18). A substantial body of research has examined the geography of crime against women in India using a range of analytical approaches. Early spatial mapping using NCRB data established the non-uniform geographic distribution of rape (19, 20). Small-area estimation of rape risk in Uttar Pradesh (21) was followed by the use of space–time scan statistics to identify persistent district-level clustering nationally between 2011 and 2020 (19). National-level small-area estimation, grounded in the statistical case for such methods over unstable direct estimates in small populations (22), has since documented shifting relative-risk patterns and emerging high-risk clusters for crimes against women between 2020 and 2022 (16). Bayesian spatio-temporal models applied to crimes against women in Uttar Pradesh (14, 17) and Maharashtra (15) have revealed district-level trends that diverge sharply from state averages. Subsequent methodological study has shown that conventional spatial models can distort these covariate associations when spatial confounding is not addressed (23). Geographically weighted regression and NFHS prevalence data have been used to map domestic violence clusters in Northeast India (24), building on earlier studies identifying the region’s socioeconomic determinants of violence (25), as well as similar studies across North India (26). Scan statistics and comparable spatial techniques have produced evidence of similar clustering in Jammu and Kashmir (27), West Bengal (28), and Rajasthan (29). A separate strand of research applies machine learning to NCRB data, including regression-based prediction (30), fuzzy clustering (31), graph-based community detection (32), provenance analysis (33), and cluster-based zoning (34) to forecast trends and classify high-risk regions. Applied systems, such as integrated GIS and a community-response pilot at the city level, have also been reported (35).

Despite this extensive body of research, existing spatial analyses of crime against women in India have typically examined a single crime category, a single state or region, or a short study period. To our knowledge, no previous study has applied a systematic framework that distinguishes persistent from emerging hotspots and combines global and local spatial autocorrelation with directional and trend classifications to a composite measure of domestic violence spanning all Indian districts over the most recent available period. Understanding not only the geographic concentration of domestic violence but also whether that concentration is sustained, intensified, newly emerged, or diminished addresses a meaningful research gap. This gap is relevant to the geographic targeting of public health and protective service resources, including the health-sector response capacities essential for violence prevention and survivor support (36). This study addresses this gap with four research questions through a district-level spatiotemporal analysis of domestic violence categories reported in NCRB across Indian districts between 2020 and 2024. First, how did district-level rates of recorded domestic violence against women change across India between 2020 and 2024, both overall and across the five crime categories examined? Second, how did domestic violence cluster spatially across Indian districts during this period? Third, which districts consistently experienced high rates and can be classified as persistent hotspots? Fourth, which districts developed emerging high-rate spatial patterns of domestic violence over the study period?

## 2 Materials and methods

### 2.1 Study area and period

This study examines district-level recorded crimes against women in India from 2020 to 2024, using a harmonized set of 636 mainland districts. Island territories (Andaman and Nicobar Islands and Lakshadweep) and non-geographic functional units were excluded to preserve a fixed contiguity-based spatial weights matrix and consistent geography across all analyses. These island territories, together with parts of the Union Territories of Jammu and Kashmir and Ladakh that were not covered by Census 2011 district boundaries, are shown on the maps within the official national boundary for completeness but were not included in any analysis.

### 2.2 Data sources and district harmonization

District-level counts for five categories (Dowry Deaths, Abetment to Suicide, Cruelty by Husband or Relatives, Dowry Prohibition Act offenses, and PWDVA cases) were obtained from the NCRB’s “Crime in India” reports (2020–2024). District boundaries and female population data were obtained from Census 2011 (37), with population estimates for later years derived from official population projections for 2011–2036 (38).

Because district boundaries and names changed between Census 2011 and NCRB reporting in 2020–2024, a crosswalk was constructed to reconcile them. Each NCRB-reported district was matched to its corresponding Census 2011 district by name. This process accounted for spelling variation, renaming, and boundary changes. Each entry was classified by match type as aggregated into its 2011 parent district (including renamed districts, spelling variants, and post-2011 subdivisions), apportioned among the 2011 districts it covers, or excluded as a non-geographic or island unit. The full district correspondence and classification for each district are provided in Supplementary Table S1. Genuine districts, including renamed or newly subdivided districts, were retained; non-geographic units and island territories were excluded; and districts created through post-2011 subdivision were merged back into their 2011 parent district. All datasets were harmonized to this 636-district geography using a unique Census code identifier. The Census 2011 shapefile was joined to population and crime-rate data through table joins in QGIS 3.44.13. When several NCRB districts corresponded to a single parent district, their case counts were summed before rates were calculated. Where a single NCRB reporting unit covered more than one Census 2011 district (the Mumbai Commissionerate, covering Mumbai and Mumbai Suburban, and Puducherry, covering Puducherry, Mahe, and Yanam), its counts were apportioned among those districts in proportion to their projected female populations. Counts were joined to district boundaries by Census code and reconciled against the NCRB totals for each year.

### 2.3 Rate and composite index construction

To estimate each district’s female population for a given year, we first calculated a state-level projection ratio (projected total population for that year divided by the Census population). This ratio was then applied to the district’s Census 2011 female population (Census 2011 female population multiplied by the state-level projection ratio). The assumption that every district in a state grew at the same rate as the state overall was used because district-level projections were not available. Crime rates were then expressed per 100,000 female population to facilitate comparisons among districts of different sizes. We also constructed a composite Domestic Violence Index (DV Index) for each year to capture all five crime categories in a single measure rather than examining them separately. These five categories were selected because, together, they constitute the NCRB crime heads that specifically codify violence within marital or domestic relationships. Equal weighting was adopted as a deliberate, non-informative default because no agreed-upon metric exists for the relative severity of these offenses. The categories are treated as complementary indicators of a common construct—recorded domestic violence—rather than as items on a unidimensional scale; therefore, a modest proportion of variance captured by a single principal component is expected and does not undermine the composite. This index reflects cases formally registered with the police, not the true level of domestic violence, because many cases go unreported. We converted each district’s rate for each category into a *z*-score relative to that year’s national distribution and then averaged the five *z*-scores with equal weighting. Because the index is centered on the national average, a negative value indicates that a district’s rate was below that year’s average. As a sensitivity analysis, we also constructed an alternative composite index for 2020 using the first principal component (PC1) of the five standardized crime rates to assess whether hotspot classification depended on the equal-weighting assumption used in the primary DV Index.

### 2.4 Descriptive and trend analysis

To characterize overall change, we calculated the median and interquartile range (IQR) for 2020 and 2024 for each crime category and the DV Index. Percentage change was calculated when the 2020 median was non-zero, otherwise, it was reported as not calculable. Because the DV Index can take negative values, its change was assessed through direct comparison of the endpoint values rather than percentage change. District-level directional change was assessed using Mann–Kendall *S* statistic for each district’s five annual values (2020–2024). Given the small number of observations, *S* was treated descriptively as an indicator of the direction of change rather than as a significance test. For each crime category, districts were classified as Increasing (*S* ≥ 6), Decreasing (*S* ≤ −6), or No clear trend (−6 < *S* < 6), so that at least eight of the 10 pairwise comparisons had to agree in direction; for the DV Index, districts were classified by the sign of *S*: Increasing (*S* > 0), Decreasing (*S* < 0), or No clear trend (*S* = 0).

### 2.5 Spatial analysis

We constructed a spatial weights matrix for the 636 districts using queen contiguity, whereby two districts were considered neighbors if they shared a border or even a corner. This matrix was used for every year of the study. The weights were constructed using a small precision threshold to correct minor digitizing gaps between adjacent district boundaries, ensuring that every district had at least one neighbor. As a further sensitivity analysis, Local Gi* classification was repeated using a k-nearest-neighbors spatial weights specification (k = 6), based on great-circle distances between district centroids, instead of queen contiguity to assess whether hotspot identification depended on the choice of neighborhood definition. Global Moran’s I was calculated for each year, using 9,999 random permutations to test significance, to determine whether DV Index values were clustered across India as a whole. The Local Getis-Ord Gi* statistic was calculated for every district in every year using the same neighbor matrix and 9,999 permutations to find those clusters at the district level. Because the Getis-Ord Gi* statistic requires non-negative values, the DV Index, which has a mean of zero by construction, was shifted by a constant (+1) before Gi* was calculated; this transformation does not affect the permutation-based pseudo-*p*-values. A district was classified as a hotspot if its Gi* value exceeded its expected value with a pseudo-*p* value of less than 0.05, and a coldspot if its Gi* value was below its expected value with a pseudo-*p* value of less than 0.05. A fixed random seed was used for all permutation tests, and the same rule was applied consistently to every district in every year. Since Gi* significance testing involved 636 simultaneous district-level tests per year, we also applied a Benjamini–Hochberg false discovery rate (FDR) correction (*α* = 0.05) (39) to assess whether the persistent and recurrent hotspot classifications remained robust under a stricter multiple-testing adjustment. We then counted the number of years, out of five, in which each district met the hotspot criterion and sorted them into four groups. The four groups are: persistent hotspot (all 5 years), recurrent hotspot (3–4 years), sporadic hotspot (1–2 years), and not a hotspot (0 years). The Mann–Kendall S statistic on each district’s five annual Gi* values was calculated descriptively to see whether clustering was getting stronger or weaker over time. A positive S indicated strengthening clustering, and a negative S indicated weakening clustering. We defined four patterns combining this trend with each district’s hotspot history. New hotspot (a hotspot only in 2024 with no earlier hotspot years), emerging hotspot (not a hotspot in 2020, a hotspot by 2024, a strengthening trend, and at least one significant year in between), diminishing hotspot (a hotspot in 2020 that lost this status by 2024, with a weakening trend), and intensifying hotspot (a persistent or recurrent hotspot with a strengthening trend). Any district that did not fit these patterns was labeled stable/other.

### 2.6 Software

QGIS 3.44.13 was used for spatial data preparation (boundary attribute joins, final 636 district geometry) and map production. Excel was used for data cleaning, crosswalk construction, rate or index calculation, descriptive statistics, hotspot classification, and Mann–Kendall trend analysis. GeoDa 1.22.0.21 (40) was used to construct the spatial weights matrix and conduct all spatial statistical analyses (Global Moran’s I, Local Getis-Ord Gi*, and principal component analysis). Maps were produced using the World Geodetic System 1984 (WGS 84) / India National Spatial Framework Lambert Conformal Conic (NSF LCC) projection (EPSG:7755) and the official national boundary of India from the Survey of India’s *International Boundary Vector Data* (*Outline of India*, generalized at 1:16 million) (41), consistent with the Political Map of India published by the Survey of India (42). District polygons remain the Census 2011 boundaries used in the analysis, and the hotspot and coldspot categories shown on the maps were derived from the Gi* values and pseudo-*p*-values using the rule described in Section 2.5.

## 3 Results

### 3.1 Descriptive and trend analysis of crime rates

Across the study period, the median district-level rate declined for two of the five crime categories (Table 1). Dowry Deaths decreased by 37.7% (from 0.63 to 0.39 per 100,000 female population), and Abetment to Suicide decreased by 23.2% (from 0.44 to 0.34). The median rate for Cruelty by Husband or Relatives increased slightly by 2.1% (from 8.01 to 8.18), and its interquartile range widened (from 15.44 to 18.60), pointing to greater dispersion across districts. The Dowry Prohibition Act and PWDVA cases had a median district rate of zero in both 2020 and 2024, so percentage change could not be calculated for either category. Both categories have a low base rate and limited district-level registration.

**Table 1:** Global Moran’s *I* for DV Index, 2020–2024 (queen contiguity, 9,999 permutations).

Year	Moran’s *I*	Expected *I*	*z*-score	Pseudo-*p*-value
2020	0.396	−0.0016	16.45	≤0.0001
2021	0.360	−0.0016	14.98	≤0.0001
2022	0.465	−0.0016	18.70	≤0.0001
2023	0.446	−0.0016	18.05	≤0.0001
2024	0.525	−0.0016	21.34	≤0.0001

Mann–Kendall trend classification showed no clear monotonic trend in most districts across all five categories (398–620 districts, 63–97%). Cruelty had the most balanced split between increasing and decreasing districts (128 vs. 110); PWDVA had the least (12 vs. 4, with 620 showing no clear trend), consistent with its low base rate. The median declined marginally (from −0.037 to −0.053) for the composite DV Index, while the interquartile range widened (0.583 to 0.617), indicating growing dispersion across districts. Mann–Kendall classification for the DV Index showed 277 districts (43.6%) with an increasing trend, 275 (43.2%) with a decreasing trend, and 84 (13.2%) with no clear trend. District-level direction was mixed even though the national median remained comparatively stable.

### 3.2 Global spatial autocorrelation

Global Moran’s *I* showed statistically significant positive spatial autocorrelation in the DV Index across all 5 years (9,999 permutations, all *p* ≤ 0.0001; Table 2), indicating non-random spatial clustering of domestic violence across Indian districts. Values ranged from 0.360 (2021) to 0.525 (2024), well above the expected value under spatial randomness [*E* (*I*) ≈ −0.0016], with *z*-scores between 14.98 and 21.34. Moran’s *I* decreased slightly from 0.396 in 2020 to 0.360 in 2021, increased in 2022 (0.465), decreased slightly in 2023 (0.446), and reached its highest value of 0.525 in 2024. Taken together, these results show that recorded domestic violence against women in India was not randomly spread across districts between 2020 and 2024, but instead formed a pattern of spatial clustering that was stronger at the end of the period than at the start (from 0.40 in 2020 to 0.53 in 2024), although the increase was not continuous year on year.

**Table 2:** District-level Getis-Ord Gi* hotspot classification, 2020–2024 (*n* = 636).

Classification	Criteria	Districts	Percentage of total
Persistent hotspot	Significant hotspot in all 5 years	30	4.7%
Recurrent hotspot	Significant hotspot in 3–4 years	52	8.2%
Sporadic hotspot	Significant hotspot in 1–2 years	85	13.4%
Not a hotspot	Never a significant hotspot	469	73.7%

### 3.3 Persistent hotspot classification

Local Getis-Ord Gi* analysis identified statistically significant hotspots of high DV Index values in every study year, ranging from 78 in 2021 to 97 in 2024 (Figure 1). Sorting districts according to the number of years in which they met the hotspot criterion (Gi* above its expected value, pseudo-*p* < 0.05) showed that 30 districts (4.7%) qualified as persistent hotspots, 52 (8.2%) as recurrent hotspots (Table 3, Figure 2). Together, these 82 districts (12.9%) showed a sustained, consistent concentration of domestic violence clustering across most or all of the study period. A further 85 districts (13.4%) were sporadic hotspots, while the majority (469 districts or 73.7%) never met the criteria. Persistent and recurrent hotspot districts formed a contiguous belt across northern and central India, mainly in Uttar Pradesh, Madhya Pradesh and Haryana, with smaller clusters in Telangana (including Hyderabad, Nalgonda and Medak), Kerala, and Bihar and Jharkhand; hotspots in Northeastern India were sporadic only (13 districts in Assam and one in Meghalaya) (Figure 2). This geographic pattern is consistent with the significant global clustering identified through Global Moran’s *I*. Applying the Benjamini–Hochberg correction substantially reduced the number of significant districts. FDR-significant hotspots numbered 4, 5, 26, 21, and 30 for 2020 to 2024, respectively, and coldspots numbered 37, 42, 58, 52, and 69. No district remained a significant hotspot in all 5 years after correction, so none met the persistent hotspot criterion under this adjustment. Of the 30 persistent hotspots, 20 (66.7%) retained significance in at least 1 year (8 recurrent and 12 sporadic under the corrected threshold), whereas 10 (33.3%) had no significant year. Among the 52 recurrent hotspots, 21 (40.4%) retained at least one significant year and 31 (59.6%) did not. The five-of-five-year criterion is therefore not robust to a conservative multiple-testing adjustment. However, the correction affected the early years most strongly. From 2022 onward, 21 to 30 districts remained significant hotspots each year, suggesting that a smaller core of districts withstands the stricter threshold. We also checked how sensitive this classification was to the equal-weighting choice. Using the first principal component (PC1) of the five standardized 2020 crime rates as an alternative index, PC1 explained only 25.6% of total variance (eigenvalue 1.28), and three components had eigenvalues above 1. Loadings on PC1 ranged from −0.490 for Dowry Prohibition Act offenses to 0.631 for Abetment to Suicide, with Cruelty by Husband or Relatives at 0.589, indicating that the five categories do not collapse into a single dominant pattern. Applying the same Gi* procedure on the PCA-weighted index identified 135 significant hotspots in 2020, compared with 91 under the equal-weighted DV Index; 55 of the 91 (60.4%) were also identified under the PCA-weighted index. Hotspot detection was therefore only moderately robust to the choice of weighting, reflecting the fact that the two indices are constructed differently. As a further sensitivity check, the classification was repeated using a *k*-nearest-neighbors (*k* = 6) spatial weights specification instead of queen contiguity. Of the original 30 persistent hotspot districts, 22 (73.3%) remained persistent, a further 4 (13.3%) were classified as recurrent, 3 (10.0%) as sporadic, and 1 (3.3%) as not a hotspot. Overall, 26 of 30 districts (86.7%) retained at least recurrent hotspot status under this alternative weights specification, indicating the core hotspot geography identified in this study is not substantially dependent on the choice of spatial neighborhood definition.

**Figure 1:**
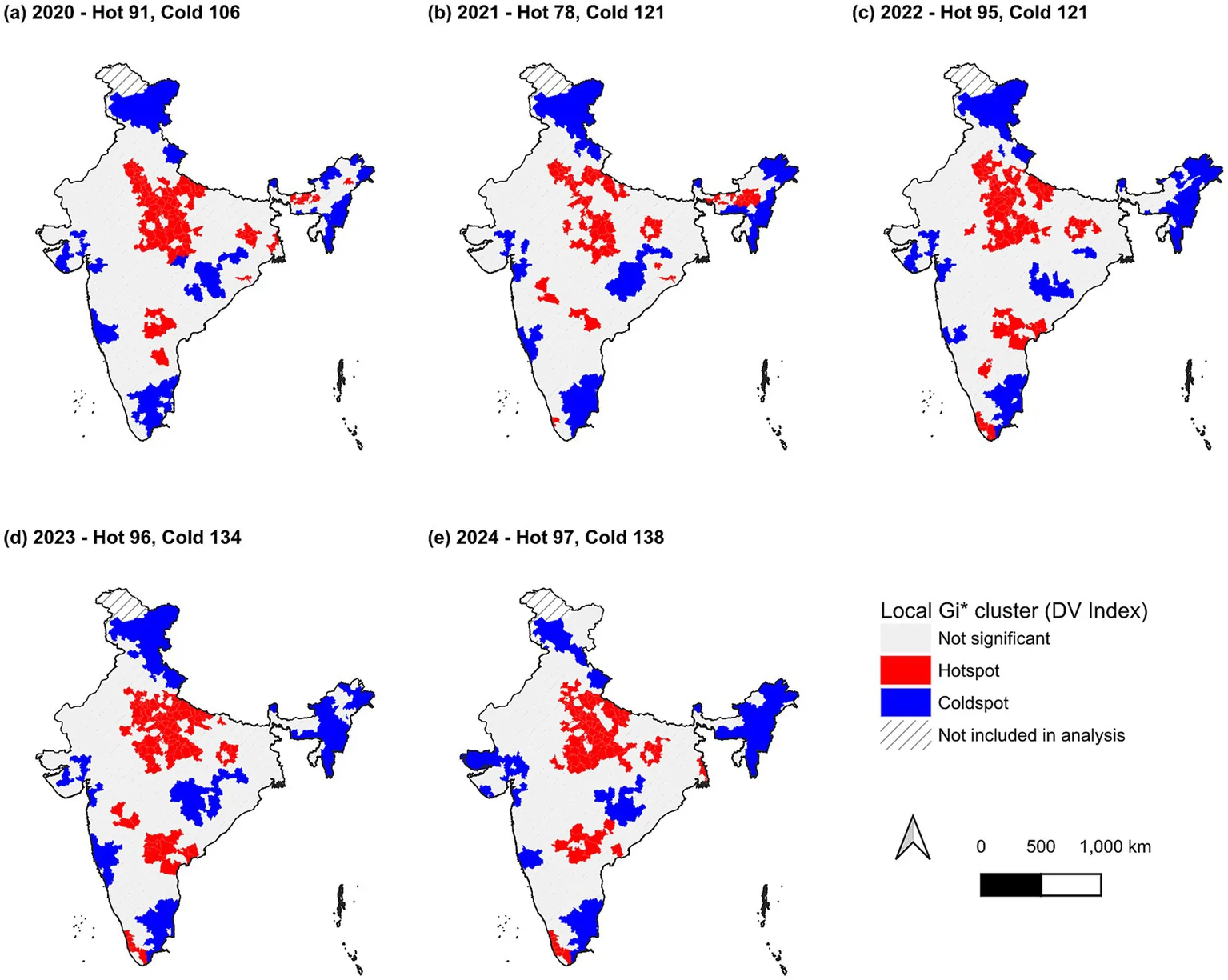
Local Getis-Ord Gi* cluster maps of DV Index by year, 2020–2024: **(a)** 2020, **(b)** 2021, **(c)** 2022, **(d)** 2023, **(e)** 2024. Hotspot: Gi* above its expected value and pseudo-*p* < 0.05; coldspot: Gi* below its expected value and pseudo-*p* < 0.05. Panel labels show the number of hotspot and coldspot districts in each year. The international boundary of India follows the Survey of India (41, 42). Hatched areas lie within the national boundary of India but outside the 636-district study geography and were not analyzed.

**Table 3:** Directional hotspot typology, 2020–2024 (*n* = 636).

Category	Criteria	Districts	Percentage of total
New hotspot	Hotspot only in 2024, none in earlier years	14	2.2%
Emerging hotspot	Not a hotspot in 2020; hotspot in 2024; positive trend, hotspot in ≥1 intervening year	31	4.9%
Intensifying hotspot	Persistent/recurrent, positive Gi* trend	38	6.0%
Diminishing hotspot	Hotspot in 2020; not hotspot in 2024; negative trend	33	5.2%
Stable/other	All remaining districts	520	81.8%

**Figure 2:**
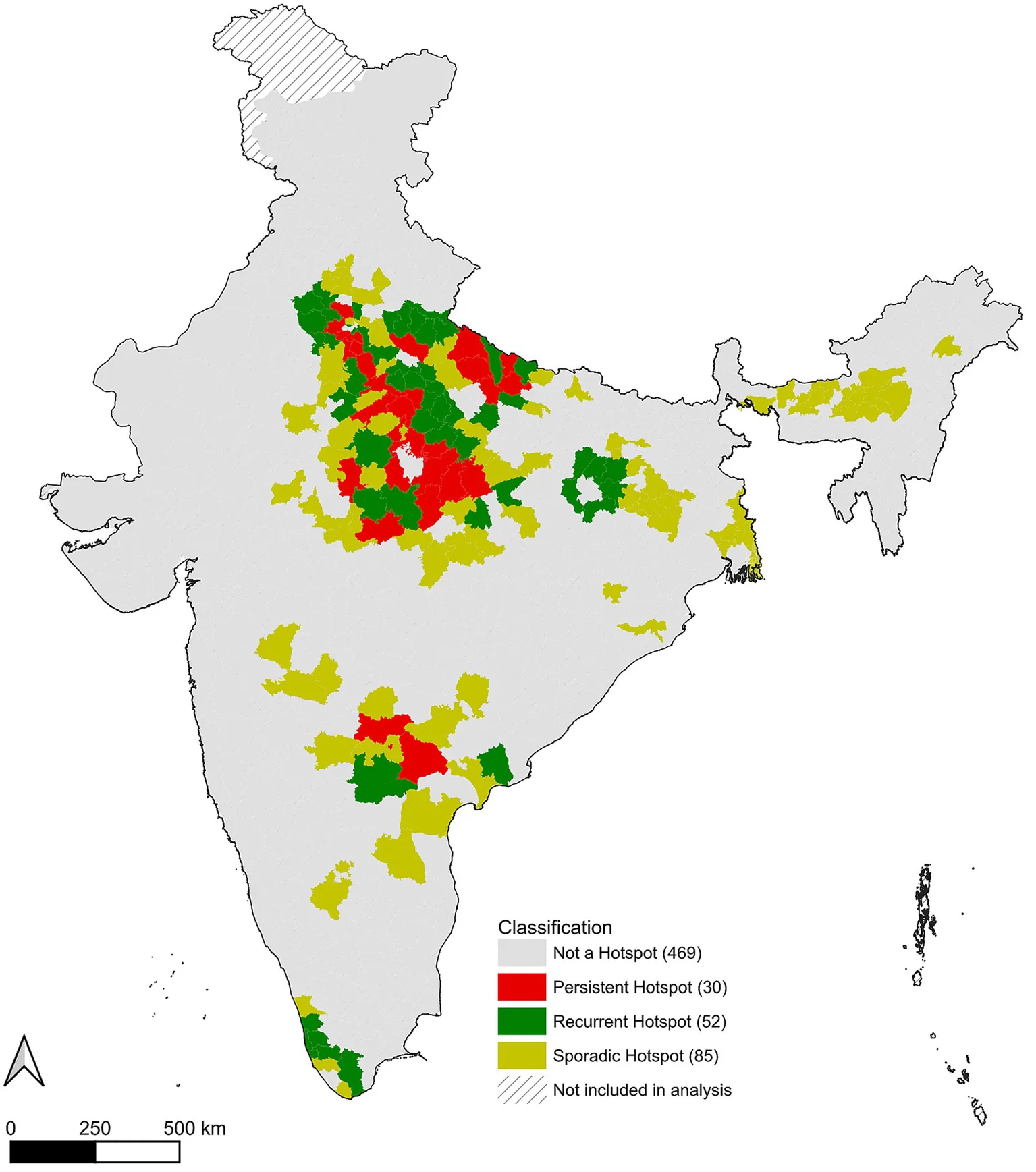
District-level persistent hotspot classification of domestic violence against women, India, 2020–2024. Districts are classified by the number of years (out of five) in which they met the hotspot criterion (Gi* above its expected value and pseudo-*p* < 0.05): persistent (5 years), recurrent (3–4 years), sporadic (1–2 years), not a hotspot (0 years). The international boundary of India follows the Survey of India (41, 42). Hatched areas lie within the national boundary of India but outside the 636-district study geography and were not analyzed.

### 3.4 Emerging hotspot typology

Combining Mann–Kendall trend analysis of each district’s five annual Gi* values with its hotspot trajectory identified four distinct patterns of directional change in local clustering between 2020 and 2024 (Tables 4, 5, Figure 3). Fourteen districts (2.2%) were classified as new hotspots, reaching significance for the first time in 2024, with no earlier clustering. Another 31 (4.9%) were emerging hotspots: not significant in 2020, significant by 2024, with a rising Gi* trajectory and at least one significant intervening year. The requirement of at least one significant intervening year for the emerging hotspot category was meant to separate districts showing a real, developing pattern of clustering from districts that simply turned significant in the very last year (the new hotspot category). We also tested a stricter version of the intensifying hotspot rule, requiring a Gi* trend statistic of 4 or higher rather than any positive value. This stricter cutoff produced a smaller number of intensifying hotspots—29 districts (15 persistent and 14 recurrent)—limited to districts with the steeper trends, compared with 38 districts under the criterion used in the main analysis. We kept the less strict rule because it avoids picking an arbitrary cutoff point, while still requiring a consistently positive trend for a district to count as intensifying. Dropping the intervening-year requirement for emerging hotspots would reclassify the 13 new hotspot districts with a positive Gi* trend as emerging (44 emerging hotspots and one new hotspot), with no change to any other category; the requirement therefore affects only the boundary between these two categories. Within the 82 persistent or recurrent hotspots, 38 (20 persistent and 18 recurrent) showed a positive Gi* trend and were classified as intensifying hotspots. A further 23 recurrent districts met the emerging hotspot criteria (the other 8 emerging hotspots were sporadic), and 7 were classified as diminishing. The remaining 14 (10 persistent and four recurrent) were placed in the stable/other category. Of the 10 persistent districts in this group, 5 showed no directional change in Gi* (*S* = 0) and 5 showed a negative trend. In these districts, local clustering weakened even though they remained significant hotspots every year. Because a persistent hotspot is significant in 2024 by definition, it cannot meet the diminishing criterion, and the absence of persistent districts from that category should not be read as independent evidence of entrenchment. Diminishing hotspots came mainly from sporadic districts (*n* = 26) and recurrent districts (*n* = 7). In total, 520 districts (81.8%), including these 14, showed no clear directional pattern and were classified as stable/other.

**Table 4:** District-level rate trends, 2020–2024 (*n* = 636).

Category	Median 2020	Median 2024	IQR 2020	IQR 2024	Percentage change	Increasing trend	Decreasing trend	No clear trend
Dowry Deaths	0.63	0.39	1.38	1.10	−37.7%	21	162	453
Abetment to Suicide	0.44	0.34	1.17	0.93	−23.2%	31	126	479
Cruelty by Husband or Relatives	8.01	8.18	15.44	18.60	+2.1%	128	110	398
Dowry Prohibition Act	0.00	0.00	0.47	0.34	NA	27	61	548
PWDVA	0.00	0.00	0.00	0.00	NA	12	4	620
DV Index	−0.04	−0.05	0.58	0.62	NA^a^	277	275	84

a Endpoint comparison reported instead of % change (Section 2.4).

**Table 5:** Decision rules for directional typology hotspot.

Category	2020 hotspot status	2024 hotspot status	Intervening years	Gi* trend (MK_S)
New hotspot	Not Significant	Significant	Not Significant (2021–2023)	–
Emerging hotspot	Not Significant	Significant	Significant ≥ 1	Positive
Intensifying hotspot	Persistent or recurrent	Persistent or recurrent	–	Positive
Diminishing hotspot	Significant	Not significant	–	Negative
Stable/other	All remaining districts			

**Figure 3:**
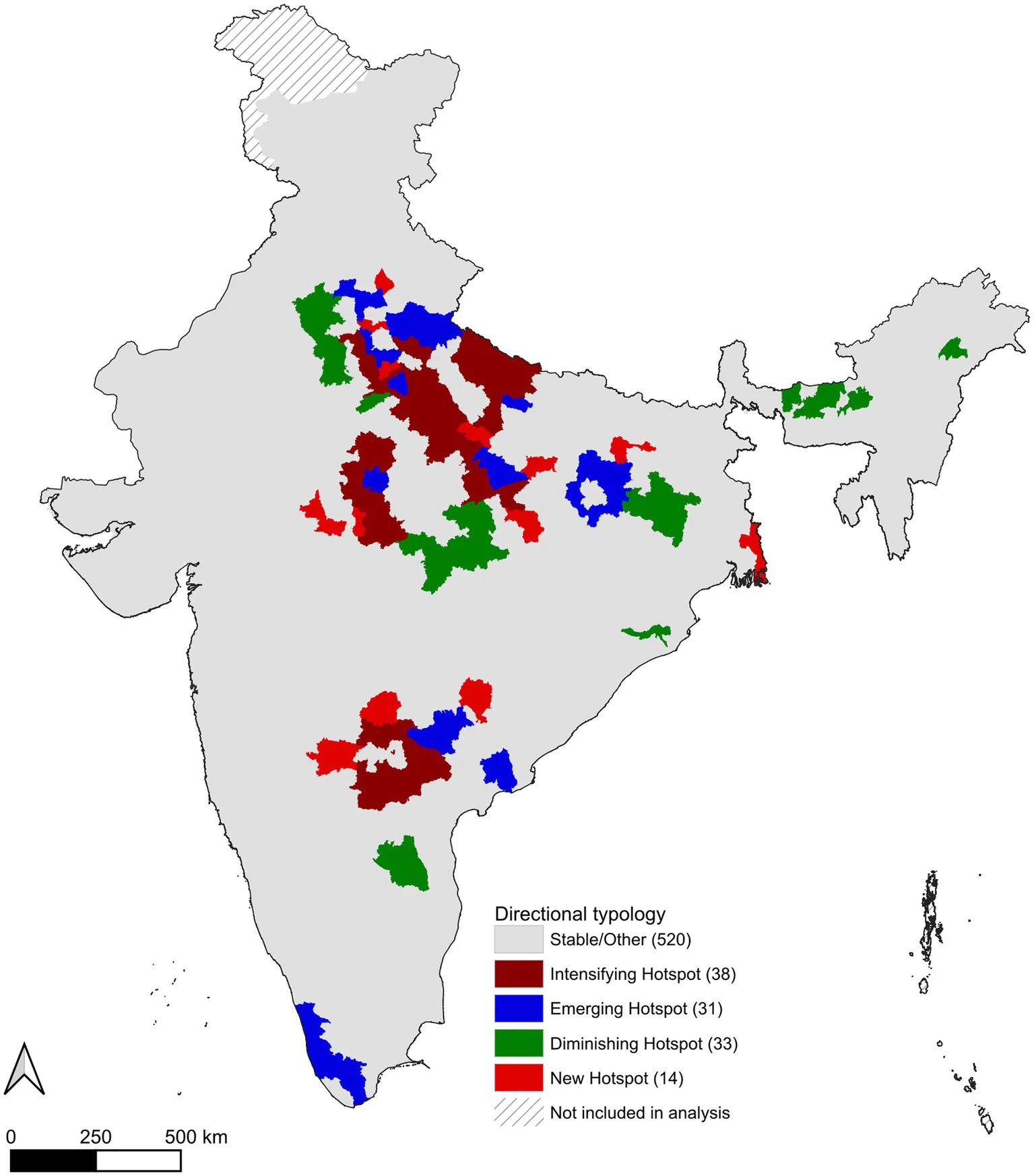
Directional hotspot typology of domestic violence against women in India, 2020–2024. Categories combine each district’s hotspot history with the Mann–Kendall trend in its annual Gi* values (decision rules in Table 5). The international boundary of India follows the Survey of India (41, 42). Hatched areas lie within the national boundary of India but outside the 636-district study geography and were not analyzed.

## 4 Discussion

This study found that recorded domestic violence against women in India was not randomly distributed across districts between 2020 and 2024. Instead, statistically significant spatial clustering was observed in every year, and clustering was stronger at the end of the period than at the beginning. Global Moran’s *I* was 0.40 in 2020 and reached its highest value, 0.53, in 2024, although the increase was not continuous, with decreases in 2021 and 2023. National median rates for most individual crime categories stayed broadly stable or declined over the period but this stability masked substantial heterogeneity at the district level. One possible explanation for the decrease in clustering strength between 2020 and 2021 is pandemic-related disruption to reporting rather than a genuine reduction in risk. This study did not directly test reporting disruption, so this remains a hypothesis rather than a confirmed finding. Global evidence links COVID-19 restrictions to sharp declines in recorded crime, followed by post-restriction rebounds (43, 44). In India, an analysis of complaints received by the National Commission for Women found that domestic violence complaints rose sharply in districts with the strictest lockdown rules and remained elevated a year later (45). Qualitative evidence from urban India suggests the pandemic worsened the underlying conditions of domestic violence (46). Local analysis identified 82 districts (12.9%) as persistent or recurrent, 31 as emerging and 38 as intensifying hotspots, showing continued or newly developing spatial concentration. By definition, persistent hotspots remained significant every year. However, 5 of the 30 showed a weakening Gi* trend, and another 5 showed no change.

These findings broadly agree with the wider literature on crime against women hotspot analysis in India and build on it. Similarly, persistent rape clustering has been documented nationally, with 5.8% of districts classified as persistently high risk between 2011 and 2020 (19). Even with different methods, crime types, and study periods, that figure is close to this study’s 4.7% persistent hotspot rate. Pooja et al. identified significant hotspots of crime against women in parts of Telangana, alongside Rajasthan, Madhya Pradesh, Haryana, and Odisha, for 2020–2022 (16). This study corroborates that finding independently and classifies Hyderabad, Nalgonda, and Warangal as intensifying or emerging hotspots. At the district level, Aligarh and Shrawasti were identified as showing rising dowry-death risk in Uttar Pradesh between 2001 and 2014 (17). Two decades later, this study classifies them as recurrent and persistent hotspots, respectively. This suggests the risk in these districts may have persisted well beyond this study’s 5-year window. Kheri is an exception: it remained a persistent hotspot here but declined in the earlier study. This divergence is not explained here, and it merits further investigation. Prior studies found only modest spatial correlation between different crime types. In Uttar Pradesh, the model-based correlation between the spatial risk patterns of rape and Dowry Deaths was 0.30 (95% credible interval 0.08–0.50), although their temporal trends were strongly correlated (0.82) (14). Domestic cruelty also showed a distinct spatial gradient relative to other crimes (15). This supports the use of a composite index here. It is also consistent with the comparatively weak clustering seen for cruelty when tested on its own. The sporadic hotspots in Assam are broadly consistent with NFHS-5-based findings of domestic violence clustering in Northeast India (24). The northern and central Indian belt overlaps substantially with the high dowry-death burden states identified over a decade earlier (47). This regional concentration in northern and central India mirrors patterns found in other NCRB-based analyses of socially patterned violence. Analyses of crimes against Scheduled Castes and Scheduled Tribes drawing on NCRB data similarly report concentration in Uttar Pradesh, Rajasthan, and Madhya Pradesh for one group rather than an even spread across states (48). This suggests the regional risk geography has been structurally stable for some time. Gi*-based classification of persistent and emerging hotspots also has precedent in other public health domains, including air pollution in India (49) and tuberculosis surveillance in Ghana (50).

These findings are consistent with the WHO’s ecological framework for violence prevention (1). They support treating domestic violence as a health-system concern as well as a criminal-justice one. The 82 persistent and recurrent hotspot districts identified here offer a concrete basis for directing existing protective infrastructure, such as One Stop Centres, PWDVA protection officers, and women helplines, toward areas with a sustained burden. This recommendation should not be read as equating high recorded case clustering with highest unmet need. A district’s hotspot status could reflect genuinely high underlying prevalence, strong reporting systems and police accessibility that make more cases visible, or both together. Districts likely fall into distinct groups based on which of these conditions dominates. In some, high recorded burden coincides with already-strong reporting and service systems, where further investment might reasonably focus on prevention rather than expanding access. In others, high recorded burden exists alongside weak service infrastructure, making basic protective capacity the more urgent priority. A third group shows low recorded burden that may nonetheless conceal high hidden prevalence, where limited reporting or service access obscures the true picture. Distinguishing these categories, rather than treating all hotspot districts as equivalent, would allow resources to match more precisely to what drives each district’s recorded pattern. This classification is based on an unadjusted threshold. FDR correction (Section 3.3) showed that the strict persistent hotspot criterion does not hold under a stricter adjustment, although a smaller core of districts remained significant from 2022 onward. Structural determinants may partly explain this geography. These include poverty, low education, and mid-level economic development (51–53). Higher police expenditure increases reporting without necessarily reducing crime (54). This cautions against reading future hotspot intensification as evidence of worsening conditions alone. The WHO’s COVID-19 health-sector guidance (36) offers a directly applicable model for the emerging and intensifying hotspot districts identified here. It recommends that violence-response services be maintained and adapted as essential during periods of elevated risk. Since this study relies on police-recorded data, these patterns should be read as showing that formal system engagement is concentrated. They do not show where prevalence is highest. This limitation is not specific to domestic violence. Other NCRB-based studies of caste-related crime in India face the same limitation. The regional clustering reported in those studies may reflect genuine differences in incidence, or differences between states in how consistently cases are reported and acted upon (55). The three-group framework outlined earlier in this section, which distinguishes recorded burden, hidden prevalence, and service infrastructure, should therefore guide the use of these hotspot locations in intervention planning. NFHS-5 found that 32% of ever-married women had experienced spousal violence (6). Although a prevalence estimate and an annual registration rate are not directly comparable, the contrast is stark: the median district rate for Cruelty by Husband or Relatives, the largest recorded domestic violence category, was 8.18 per 100,000 women in 2024 (Table 1). Triangulating this with survey-based prevalence estimates remains an important direction for future studies (10). Several limitations should be considered when interpreting these findings. DV Index is derived from police-recorded case data and therefore reflects case activity rather than the true prevalence of domestic violence, a known substantial gap. Fewer than 2% of women facing domestic violence ever report it to police (5), and only 14% of women who experienced physical or sexual violence sought help of any kind (6). Geographic differences in police accessibility, reporting norms, the functioning of PWDVA mechanisms such as protection officers, and wider institutional capacity may therefore partly account for the observed hotspots, independently of true levels of victimization. Global Burden of Disease estimates of suicide rates for 2015 were 19.7 and 8.9 times higher than NCRB-reported rates in Bihar and Uttar Pradesh, respectively (56). A similar undercount may affect this study’s Abetment to Suicide category, in which districts with decreasing trends outnumbered those with increasing trends (126 vs. 31); part of this apparent decline may reflect registration practices rather than a genuine fall in incidence. Because every analysis was conducted at the district level, the results show broad patterns across areas. They cannot tell us about risk to individual people or about cause and effect. This limitation matters even more because research shows that violence risk varies at a much smaller scale than the district, shaped by both wider environmental factors and conditions specific to individual streets (57). District-level female population figures for 2020–2024 also depend on one key assumption. Since no population projections existed at the district level, this study applied the same state-level growth rate to every district within that state. This assumption does not account for migration, urbanization, or differences in growth between districts within the same state. A fast-growing urban district and a slower-growing rural district in the same state could both have their true population under- or overestimated in opposite directions. Since crime rates are calculated by dividing case counts by this estimated population, any such bias would carry directly into the rates themselves, and potentially into which districts are classified as hotspots. A related assumption affects the few districts without a separate NCRB reporting unit. Counts for the Mumbai Commissionerate were apportioned between Mumbai and Mumbai Suburban, and counts for Puducherry were apportioned among Puducherry, Mahe, and Yanam, in proportion to female population. This assumes equal recorded rates across the districts covered by a single police unit, so it cannot detect true differences between them.

DV Index gives equal weight to all five crime categories. We checked this choice through a PCA-based sensitivity analysis for 2020, and hotspot classification overlapped only moderately under the alternative weighting: 60% of the original hotspots were also identified. Nevertheless, the two indices do not measure exactly the same construct. PC1 partly reflects one category trading off against another, rather than a clean, uniform signal of overall violence intensity. This is consistent with prior studies showing these categories do not share the same spatial patterns (14). The PCA check was carried out for 2020 only. In addition, because Cruelty by Husband or Relatives dominates the raw rates (median 8.01 per 100,000 in 2020, against 0.63, 0.44, 0.00 and 0.00 for the other four categories), *z*-scoring followed by equal weighting gives sparsely registered offenses the same leverage in the index as the dominant category. Mann–Kendall results were treated as descriptive signals of direction rather than statistically confirmed trends because the time series was short. Hotspot detection primarily used queen contiguity as the spatial weights specification. We also tested a k-nearest-neighbors (*k* = 6) weights specification as a sensitivity analysis. Under this alternative, 86.7% of persistent hotspot districts retained at least recurrent status. This suggests the core geographic pattern is not just an artifact of the chosen neighborhood definition. Still, a few districts (4 of 30) fell below recurrent status under this choice. More recent confounding-adjustment techniques (23) were not applied in this analysis. These represent a direction for future refinement. The pseudo-p threshold used for Local Gi* (*p* < 0.05) was unadjusted, consistent with common practice in this literature. We also applied an FDR correction as a check; the strict persistent hotspot classification did not survive it, although a small core of hotspot districts remained significant in the later years of the study period. Finally, some districts were split or newly formed after Census 2011. These districts were folded back into their original parent boundaries. This kept the geography consistent over time. It may hide variation that emerged after those later subdivisions.

## 5 Conclusion

This study provides the first district-level spatiotemporal analysis of recorded domestic violence against women across India using a composite index that integrates five crime categories. The findings show that recorded domestic violence is not randomly distributed but is concentrated in a recurring geography of hotspots, with global clustering stronger at the end of the study period than at the start, although the strict persistent hotspot classification was not robust to FDR correction. This pattern is consistent with prior spatial evidence on crime against women in India. But breaking hotspots into persistent, emerging, and intensifying categories adds a time dimension that national studies have not captured before. These findings have implications for the geographic allocation of prevention and response services such as One Stop Centres and helplines. Hotspot status should, however, be treated as one input among several, since districts with a sustained recorded burden need to be distinguished from those where low recorded rates may reflect limited reporting or weak service access. Since the analysis relies on police-recorded case data, these results should be understood as a map of formal system engagement with domestic violence rather than a direct measure of its true underlying prevalence. Survey-based prevalence data, finer spatial resolution, and formal confounding-adjustment methods would help establish whether recorded crime hotspot mapping can serve as a public health surveillance tool.

## Data Availability

Publicly available datasets were analyzed in this study. Crime data are available from the National Crime Records Bureau (NCRB), Ministry of Home Affairs, Government of India, at https://www.ncrb.gov.in (Crime in India annual reports, 2020–2024). District boundary and population data are available from the Census of India 2011, Office of the Registrar General & Census Commissioner, India, at https://censusindia.gov.in. Official population projections (2011–2036) are available from the National Commission on Population, Ministry of Health and Family Welfare, Government of India. The national boundary used for mapping is available from the Survey of India (International Boundary Vector Data, Outline of India). The derived district-level dataset, spatial weights matrix, and hotspot classification results are available from the corresponding author on reasonable request.
